# Opening the Effector Protein Toolbox for Plant–Parasitic Cyst Nematode Interactions

**DOI:** 10.1016/j.molp.2016.09.008

**Published:** 2016-11-07

**Authors:** Sebastian Eves-van den Akker, Paul R.J. Birch

**Affiliations:** 1 Division of Plant Sciences, College of Life Sciences, University of Dundee, Dundee DD1 5EH, UK; 2 The Department of Biological Chemistry, John Innes Centre, Norwich Research Park, Norwich NR4 7UH, UK

Some biotrophic plant pathogens have the remarkable ability to alter plant developmental morphology and subcellular architecture. At least three examples of this ability arose independently within the phylum Nematoda. For one such example, the cyst nematodes, plant cell manipulation is rapid and profound; within days the cell cycle of a single cell in the vascular cylinder is arrested at G2, the vacuole reduces in size and fragments, the nucleus greatly enlarges, the cytoplasm is enriched in subcellular organelles by extensive proliferation of the smooth endoplasmic reticulum, ribosomes, mitochondria, and plastids (chloroplasts and amyloplasts), and the cell wall is degraded to promote protoplast fusion in an iterative manner, ultimately incorporating hundreds of adjacent cells (Figure 1; Jones, 1981).

## Effectors: The Molecular Tools to Manipulate the Plant Cell

**Figure 1:**
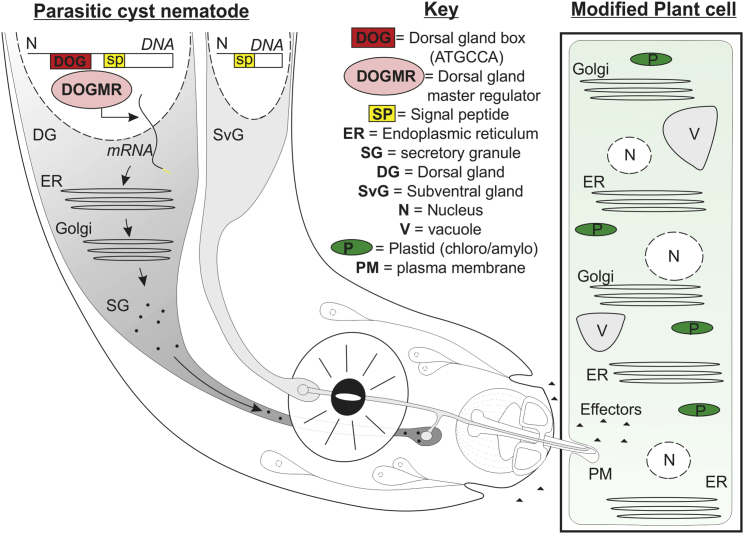
Schematic of Plant Parasitic Nematode Effector Production and Infection. Parasitic cyst nematodes contain two primary classes of gland cells (dorsal [DG] and subventral [SvG]). Dorsal gland-expressed effectors often contain a dorsal gland box (DOG box) in their promoter regions. We hypothesize that an as yet unidentified dorsal gland master regulator (DOGMR), specifically expressed in the dorsal gland cell, will recognize this motif, initiating the processes of transcription, translation, and secretion through the endoplasmic reticulum (ER) Golgi complex to secretory granules (SG). SGs release effectors (filled triangles) into the lumen of the stylet, to be delivered both in the apoplasm and across the plant plasma membrane (PM). The DOG box reveals the extended repertoire of effectors that rapidly manipulate plant development and immunity, ultimately resulting in a novel syncytial organ with reduced and fragmented vacuole (V), multiple enlarged nuclei (N), and proliferated ER, Golgi, and plastids (P).

To achieve such manipulation, parasitic nematodes, like other plant pathogens, are equipped with a set of molecular tools termed effectors, secreted molecules which manipulate the host to the benefit of the pathogen. Such effectors are either delivered directly into the apoplasm (Eves-van den Akker et al., 2014) or translocated across the host plasma membrane (Jaouannet et al., 2012) through a needle-like stylet (Figure 1). Effectors are likely central to manipulating plant development. Yet, despite recent progress in the field, the functions and even identities of most are unknown.

## The Pharyngeal Glands: The Toolbox

While not the only secretory tissues in cyst nematodes, the pharyngeal gland cells are physical compartments in which effectors are produced, and thus can be regarded as a toolbox for infection. Cyst nematodes have two sets, subventral and dorsal: the former are primarily active while the nematode migrates through host tissue, while the latter are primarily active during the sedentary parasitic stages (Endo, 1987). Given the importance of effectors in determining plant developmental fate, recent years have seen considerable effort devoted to identifying the genes expressed within these gland cells.

## The DOG Box: Finding the Tools for the Job

Plant pathogens, parasites, and symbionts secrete effectors. While many effector proteins are thought to encode a secretion signal, not all secreted proteins are effectors. This selective delivery of proteins necessitates a mechanism to differentiate those secreted proteins that manipulate the plant cell (the effectors) from those that do not. Given that effectors are a minority of secreted proteins within an organism, the default state for a secreted protein cannot be effector. Researchers studying plant pathogenic microbes must therefore distinguish effectors from within a considerable secretome. In *Phytophthora*, a solution to this problem has apparently manifested as a protein-coding, RXLR, motif (Whisson et al., 2007). The RXLR motif is a good predictor of effectors (Win et al., 2007) and has facilitated rapid advances in understanding by allowing *in silico* prediction of the effector repertoire.

For cyst nematodes, we have been unable to identify a similar or equivalent motif in the protein-coding region of effectors (Eves-van den Akker et al., 2016). This apparent distinction can be reconciled by the presence of the highly specialized gland cells as a physical compartment that, during infection, is essentially dedicated to producing and trafficking cargo for *in planta* delivery. We predict such a highly specialized tissue would require a transcriptional master regulator (termed here the dorsal gland master regulator [DOGMR], Figure 1) that itself is specific to the dorsal gland cell and coordinates the expression of effectors and packaging machinery by recognition of a dorsal gland box (DOG box).

Consistent with this, Eves-van den Akker et al. (2016) identified a putative dorsal gland promoter element. This 6-bp DOG box, of the canonical motif ATGCCA, is highly enriched ∼150 bp upstream of the coding start site in the promoter region of known dorsal gland effectors. More than 77% of known dorsal gland effectors contain at least one DOG box (average ∼2.5/500 bp promoter), with representatives from 26 of the 28 experimentally validated dorsal gland cell effector families (Eves-van den Akker et al., 2016). Importantly, genes with more DOG boxes in their promoter regions were more likely to encode proteins with a signal peptide for secretion, a required feature of an effector. Exploiting the DOG box for utility, Eves-van den Akker et al. (2016) predicted a superset of putative effectors associated with this promoter motif, experimentally validated gland cell expression in two novel genes by *in situ* hybridization, and cataloged DOG effectors from available cyst nematode genomes.

The ability to predict dorsal gland proteins *in silico* represents a major turning point in the study of plant–nematode interactions. Given that (1) the dorsal gland is highly active in the production of secretory granules very soon after infection (3 days) when compared with other secretory glands (Endo, 1987), and (2) the vast majority of known effector proteins originate from the dorsal glands (Eves-van den Akker et al., 2016), identifying secreted proteins with DOG boxes in their promoter will likely include those that alter plant development and immunity. Interestingly, focusing on non-secreted proteins with DOG boxes in their promoter region may reveal some of the machinery used to package effectors before delivery.

## The Discovery of the DOG Box Will Serve Three-Fold

1. The trend is set to exploit the ever increasing genomic information to identify vastly more effectors than previously known by utilizing promoter motifs descriptive of other glands (subventral, amphids, etc.), other nematodes (reniform, pine-wilt, root-knot, etc.), or indeed other animals (aphids, etc.). Given the almost complete lack of overlap in effector repertoires between nematodes with independent evolutionary origins of plant parasitism (Eves-van den Akker et al., 2016), expanding these *in silico* analyses will likely reveal many novel effectors and effector functions, ultimately leading to a better understanding of how these nematodes modify plant development and immunity.
2. If we knew the details of how these effectors function, we could exploit their actions for utility. Exploiting plant-pathogen effector functions has been, and continues to be, influential in many scientific fields: *Agrobacterium*-mediated transformation and TALEN genome editing (Bedell et al., 2012) are good examples of this utility. The DOG box moves us one step closer to identifying the plant development- and immunity-altering toolbox of parasitic cyst nematodes. The ability to increase chloroplast, amyloplast, or mitochondrion number in discrete tissues has clear potential for biotechnological utility.
3. Identification of DOGMR, the predicted master regulator that binds these motifs, could open new avenues for disease control. The feasibility of host-induced gene silencing (HIGS) has been demonstrated for a number of plant pathogens/parasites (nematodes [Huang et al., 2006], aphids [Pitino et al., 2011], etc.). Master regulators, such as DOGMR, present attractive targets for a HIGS approach. Removing the switch that activates the expression of all effectors would undoubtedly undermine the infection process.

The DOG box represents a step-change in our fundamental understanding of how nematodes have evolved to manipulate plant development and immunity. This advance may catalyze the development of novel control strategies within plant–nematode interactions and lead to potential biotechnological utilities beyond plant–nematode interactions.

## Funding

S.E.-v.d.A. is supported by BBSRC grant BB/M014207/1.
